# Prognostic microRNAs in upper tract urothelial carcinoma: multicenter and international validation study

**DOI:** 10.18632/oncotarget.17884

**Published:** 2017-05-16

**Authors:** Laura Izquierdo, Ruth Montalbo, Mercedes Ingelmo-Torres, Carme Mallofré, Miguel Ramírez-Backhaus, Jose Rubio, Antoine G. Van der Heijden, Ewout Schaafsma, Antonio Lopez-Beltran, Ana Blanca, Nathan Lawrentschuk, Antonio Alcaraz, Lourdes Mengual

**Affiliations:** ^1^ Department of Urology, Hospital Clinic, IDIBAPS, Barcelona, Spain; ^2^ Laboratory of Urology, Hospital Clinic, IDIBAPS, Barcelona, Spain; ^3^ Department of Pathology, Hospital Clinic, Barcelona, Spain; ^4^ Department of Urology of Fundación IVO, Valencia, Spain; ^5^ Department of Urology, Radboud University Centre, Nijmegen, Netherlands; ^6^ Department of Pathology, Radboud University Centre, Nijmegen, Netherlands; ^7^ Department of Pathology of Reina Sofía Hospital and Maimonides Biochemical Research Institute, Córdoba, Spain; ^8^ Department of Urology, Maimonides Biochemical Research Institute of Córdoba, Córdoba, Spain; ^9^ Department of Urology, University of Melbourne, Department of Surgery/Olivia Newton-John Cancer Research Institute, Melbourne, Australia

**Keywords:** microRNAs, prognosis, upper tract urothelial carcinoma

## Abstract

**Objective:**

To validate previously discovered miRNAs (miR-31-5p and miR-149-5p) as prognostic factors for UTUC in an independent cohort of UTUC patients.

**Patients and Methods:**

Multicenter, international and retrospective study of formalin-fixed paraffin-embedded tissue samples from 103 UTUC patients (45 progressing and 58 non-progressing) who underwent radical nephroureterectomy. Total RNA was isolated and reverse transcribed. The expression of target miRNAs (miR-31-5p and miR-149-5p) and the endogenous control miR-218-5p was evaluated in all samples by reverse transcription quantitative PCR. Normalized miRNA expression values were evaluated by multivariate forward stepwise Cox regression analysis. Kaplan Meier curves were used to discriminate between two groups of patients with a different probability of tumour progression.

**Results:**

The mean age (range) of the series was 67 (33-94) years. Overall, 45 patients (43.7%) developed tumour progression and 32 patients (31.2%) died, 20 of these (62.5%) due to their UTUC, after a median follow-up of 36 months. The mean time for tumour progression and cancer-specific survival were 15 and 20 months, respectively. Five year tumour progression free survival and cancer-specific survival were 58% for ≤ pT2, 36% for pT3 and 0% for pT4 and 67.8% for ≤ pT2, 50.6% for pT3 and 0% for pT4, respectively. In the multivariate analysis, expression of miR-31-5p was found to be an independent prognostic factor of tumour progression (HR 1.1; 95% CI 1.039-1.273; p=0.02). Kaplan Meier curve shows that miR-31-5p expression values are able to discriminate between two groups of UTUC patients with a different probability of tumour progression (p=0.007).

**Conclusions:**

We have been able to validate our previous results in an independent multicentre international cohort of UTUC patients, suggesting that miRNA-31-5p could be a useful prognostic marker of UTUC progression. The application of miRNA expression values to clinical practice could refine the currently used clinicopathological-based approach for predicting UTUC patients’ outcome.

## INTRODUCTION

Upper tract urothelial carcinomas (UTUCs) are uncommon and account for only a small number (5-10%) of urothelial carcinomas. The majority of UTUCs are invasive at diagnosis compared to bladder tumours having only up to a quarter invasive at presentation[1, 2]. Due to the aggressive nature of UTUC, radical nephroureterectomy (RNU) remains the “gold standard” treatment for localized tumours [3].

Established prognostic factors associated with tumour progression and survival are pathological stage and tumour grade. However, they are insufficient to predict the individual outcome for UTUC patients [4, 5]. A greater understanding of the biological behaviour of tumours would allow individualized medicine, in an attempt to decrease morbidity and importantly improve survival. microRNAs (miRNAs) are a family of short (average of 22nt long), naturally occurring, small antisense non-coding RNAs, that have emerged as important post-transcriptional regulators of gene expression [6]. They have wide distribution as endogenous controllers of gene expression by binding to the 3’-untranslated region of specific miRNAs [7, 8]. As miRNAs may regulate a significant portion of the transcriptome and proteome, considerable attention has focused on miRNAs as mediators or biomarkers of disease [9, 10]. miRNAs are critical to cancer pathogenesis, as they can act as oncogenes or tumor suppressor genes. They may also have potential value as prognostic or diagnostic biomarkers in the clinical setting [11]. Many cancers have had dysregulation of miRNAs documented [12–14], including urothelial tumours such as bladder cancer [15] and UTUC [16, 17].

We have previously examined the miRNA expression pattern of UTUC tissue samples from progressing and non-progressing patients in order to identify putative miRNAs that may be used as prognostic markers in UTUC. miRNA-31-5p and miRNA-149-5p have been identified as prognostic factors of tumour progression and cancer specific survival [17]. In the present study, we aim to retrospectively validate miRNA-31-5p and miRNA-149-5p as prognostic markers of UTUC in a multicenter cohort of UTUC patients.

## RESULTS

### Patient characteristics

The final cohort consisted of 103 patients. Median age (range) of the cohort was 67 (33-94) years. Gender balance favored men 83:20. The median (range) follow-up oin this series was 36 (6-384) months. The patients’ histopathological characteristics are outlined in Table 1.

**Table 1:** Histopathological features of patients with UTUC

		Radboud university medical centre Nijmegen	Instituto Valenciano de Oncología	Hospital Universitario Reina Sofía de Córdoba	Hospital Clínic de Barcelona	Total (%)
**Patients**		**36**	**22**	**5**	**40**	**103 (100)**
**Gender (male/female)**		31/5	18/4	4/1	30/10	**—**
**Median age**		64	67	74	73	**—**
**Histological Grade**	**I**	1	8	2	2	13 (12.6)
	**II**	9	10	0	18	37 (35.9)
	**III**	26	4	3	20	53 (51.5)
**Pathological Stage**	**pTa**	8	4	1	8	21 (20.4)
	**pT1**	4	2	1	8	15 (14.6)
	**pT2**	5	8	1	7	21 (20.4)
	**pT3**	11	8	2	12	33 (32)
	**pT4**	6	0	0	5	11 (10.7)
	**pTis**	2	0	0	0	2 (1.9)
	**N+**	5	7	0	8	20 (19.4)
	**M+**	2	3	0	1	6 (5.8)

**Figure 1:**
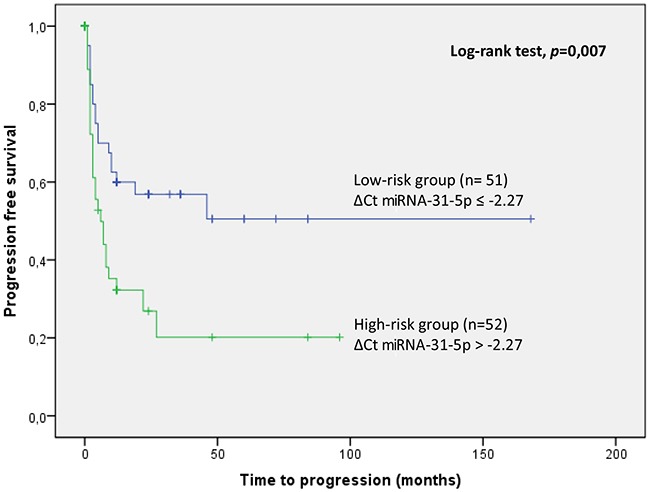
Kaplan Meier curve of tumor progression according to miRNA-31-5p expression values

Overall 45 patients (43.7%) recorded progression of their tumours at a median follow-up of 36 months. Tumour progression occurred at a median of 15 (2-168) months. By pathological stage, one and five year tumour progression free survival were 80% for ≤ pT2, 76% for pT3 and 45% for pT4 and 58% for ≤ pT2, 36% for pT3 and 0% for pT4, respectively (p<0.05) (Figure 1).

During follow-up, 32 patients (31.2%) died, 20 of them (62.5%) due to their UTUC. One was pTa (5%), two were pT1 (10%), three pT2 (15%), eight pT3 (40%) and six pT4 (30%). Disease other than UTUC accounted for mortality in twelve patients. The median (range) time to cancer-specific death was 20 (9-85) months. Based on pathological stage, the one and five year cancer-specific survival were 96.5% for ≤ pT2, 65.7% for pT3 and 40% for pT4 and 67.8% for ≤ pT2, 50.6% for pT3 and 0% for pT4, respectively (p<0.05).

### Correlations between clinical features and miRNA expression

We found that histological grade correlated with pathological stage (R=0.53, p=0.001). In addition, miRNA-31-5p correlated with histological grade (R=0.21, p=0.03) and pathological stage (R=0.29, p=0.002). Non-significant correlations were found between miRNA-149-5p with histological grade (p=0.589) and pathological stage (p=0.495).

### Association of miRNA expression with progression and survival

The expression pattern of miR-31-5p and miR-149-5p are provided in Supplementary Figure 1 of supplementary material. To verify whether these two selected miRNAs (miR-31-5p and miR-149-5p) were independent prognostic factors of patients’ progression and survival, the miRNAs expressions were analyzed using a Cox regression model. Univariate analysis including miRNA-31-5p and miRNA-149-5p showed that the expression of miRNA-31-5p was an independent prognostic factor of tumour progression (HR=1.107; 95% CI 1.008-1.217, p=0.033 and HR=0.998; 95% CI 0.831-1.199, p=0.985, respectively). Multivariate regression analysis demonstrated that expression of miRNA-31-5p was an independent prognostic factor of tumour progression (HR=1.1; 95% CI 1.039-1.279, p=0.02). Contrarily, the two miRNAs were not associated with cancer-specific survival.

miRNA-31-5p expression values are able to discriminate between two groups of UTUC patients with a different probability of tumour progression (p=0.007) using Kaplan Meier curves. UTUC samples with miRNA-31-5p expression values (ΔCt) greater than the cutoff value (-2.27) have a greater risk of tumour progression (high-risk group) and samples with miRNA-31-5p expression values lower/equal than the cutoff value have low risk tumour progression (low risk group) (Figure 1).

### Target prediction and functional enrichment of miR-31-5p

The DIANA-miRPath miRNA analysis, with miRNA-31-5p, shows several statistically significant predicted Kyoto Encyclopedia of Genes and Genomes (KEGG) terms related with the adherens junction, TNF signalling pathway, cGMP-PKG signalling pathway among others (Supplementary Table 1 of supplementary material).

## DISCUSSION

The prognosis of UTUC remains unpredictable in many instances. Approximately one third of UTUC patients die from their tumour after five years of follow-up and the commonly utilised prognostic factors are unable to accurately predict individual tumour behaviour [18]. Therefore predicting disease progression in a specific patient remains a challenge. Several molecular prognostic biomarkers have been previously investigated in UTUC patients [19–27], but have not transitioned to becoming clinically valuable tools as yet.

Identification of new prognostic tools is of great interest in UTUC to adapt treatments according to the molecular risk of a particular tumour. If we could identify a patient with a high risk of progression (clinical, molecular or both), it would be possible to increase surveillance intervals and/or consider additional treatments such as chemotherapy. Experimental work has shown that miRNAs are dysregulated in most cancer types and have demonstrated significant diagnostic and prognostic value in different malignancies [28–31]. In the case of UTUC, there are only a few studies evaluating miRNAs as diagnostic biomarkers. Kriebel *et al*. reported that miRNA expression is altered in UTUC and showed that circulating miRNA-141 may be useful as diagnostic biomarker [32]. Tao *et al*. identified a ten-serum miRNA-based expression profile that was useful in discriminating between UTUC cases and controls [16].

However, as far as we could ascertain, our group was the first to explore the potential prognostic value of miRNA expression profiles in UTUC patients [17]. Using a global miRNA profiling of UTUC samples, we identified 26 miRNAs differentially expressed within tissue samples from patients with progressive and non-progressive UTUC. After validation of such miRNAs using an independent multicenter cohort, we were able to identify miRNA-31-5p and miRNA-149-5p as independent predictors of tumour progression. Here, we report the validation of these two previously discovered prognostic miRNAs in an independent international multicenter series of UTUC patients. As demonstrated, miRNA-31-5p expression predicts tumour progression in this independent cohort. Hence miRNA-31-5p discriminated between two groups with a highly significant differential probability of tumour progression. Thus, miRNA-31-5p provides a biomarker to identify a sub-group of patients with a poorer prognosis and may help redefine the current clinicopathological approach making it a valuable clinical tool.

In addition, we found a statistical correlation between miR-31-5p with the most established prognostic factors in UTUC, histological grade and pathological stage.

miRNA-31-5p has been previously reported to be upregulated in colon, lung and cervical cancer [33–35]. On the other hand, miRNA-31-5p was found down-regulated in bladder, prostate, gastric, breast and serous ovarian cancer [36, 37]. These data suggested that miRNA-31-5p can behave either as tumour suppressor or as an oncogenic miRNA, which may be dependent on the cancer type. In concordance with our previous results, we found a down-regulation of miRNA-31-5p in progressive UTUC patients [17]. Interestingly, Wang et al. revealed that miRNA-31-5p was down-regulated in bladder cancer patients with an unfavourable outcome [38]. The miRNA-31-5p gene is located on 9p21.3. DNA lost at 9p21 has been frequently observed in bladder and UTUC patients [39, 40]. Across differing cancer types, genetic variants of the 9p21 region have been associated with the development of multiple cancers which suggests this region's importance [41]. Therefore, downregulation of miRNA-31-5p is in accordance with the loss of this chromosomal region in urothelial cancers. Moreover, in support of our study, it has been reported that suppression of miRNA-31-5p enhances chemotherapy effectiveness (5-fluoracil) at earlier stages, and additionally interferes with cell migration and invasion of colon cancer cells [37].

Furthermore, analysis of KEGG pathways corroborates that miRNA-31-5p is biologically meaningful. Several possible signalling pathways, such as the adherens junction, TNF signalling pathway, cGMP-PKG signalling pathway were predicted to be modified by miRNA-31-5p. Among others, the adherens junction contributes to cells physical linking and regulating of cell–cell contacts both critical for tissue and organ morphogenesis and remodeling. TNF is a pro-inflammatory cytokine that plays a crucial role across a range of cellular activities from proliferative through to apoptotic events. TNF has also been identified in cell dysregulation that leads to disease making it a target of interest for molecular study. It appears that many signal pathways are involved which lead to the observed TNF-mediated biological responses [42]. cGMP-PKG is implicated in the regulation of cell division and synthesis.

One of the potential strengths of this study is the methodology whereby we were able to use archival FFPE samples to obtain miRNA expression patterns. This has implications for clinical practice due to the ability to quickly translate results from “bench-to-bedside”. Moreover, this study represents a multicenter international cohort with long-term follow-up, making the data more generizable than in a single institutional cohort. There are some limitations. Firstly, although multi-center, the final number of patients studied may still be considered insufficient. Secondly, in the pursuit of in robust data, a cutoff of RNA quality was used, and a high percentage of samples were excluded from the analysis. Although the methodology reported could be refined in future studies, we maintain that we have a reliable prognostic biomarker for UTUC patients. Thirdly, although we found miRNA-31-5p down-regulated in progressing patients, the literature supports the clinical value of down-regulated biomarkers in cancer [43]. As such, we strongly believe that our results are clinically applicable.

In conclusion, the current results support the validation of our previous finding in an independent cohort, confirming that miRNA-31-5p is a discriminator between two groups of UTUC patients having differeing probabilities of tumour progression. Ultimately being able to identify new miRNAs associated with tumour progression in UTUC patients will allow individualized medicine with tailored treatment and surveillance strategies.

## PATIENTS AND METHODS

### Patient population

A retrospective study in which a total of 103 patients with UTUC who underwent nephroureterectomy in 4 different centres (Radboud university medical centre Nijmegen, Netherlands, Instituto Valenciano de Oncología-Spain, Hospital Universitario Reina Sofía of Córdoba-Spain, Hospital Clínic of Barcelona-Spain) between 1990 and 2012 were included. The only exclusion criterion was lack of tissue from the archive blocks. Histopathological characteristics of the UTUC patients are shown in Table 1. None of the patients received neoadjuvant chemotherapy. A total of 13 patients (12.6%) received adjuvant chemotherapy. Tumours were graded and classified according to the WHO's [44] and the TNM's classification of the International Union Against Cancer [45].

Tissue samples were obtained under institutional review board-approved protocol.

All patients were followed up postoperatively at 3-month intervals for the first year, at 6-month intervals for the next 2 years, and annually thereafter. Physical examination, abdominal and pelvic CT scan, cytology and cystoscopy were used to follow these patients up.

Tumour was considered to be progressing when distant metastasis or pathological nodes developed during the follow-up.

### Tissue specimens and RNA isolation

Upon obtainment, the tissue was fixed in 10% formalin within 24h and subsequently embedded in paraffin. A slide of each specimen was stained with haematoxylin-eosin to determine the presence of tumour cells. The pathologist revised haematoxylin-eosin slides and only those paraffin blocks with a minimum of 75% of tumour cells were selected. Total RNA was isolated from specimens (80-Δm) using the RecoverAll Total Nucleic Acid Isolation Kit (Ambion INC) according to the manufacturer's protocol. Total RNA was quantified by spectrophotometric analysis at 260 nm (NanoDrop Technologies, Wilmington, DE, USA).

### miRNA validation by reverse transcription quantitative PCR (RT-qPCR)

RT-qPCR reactions were performed according to the manufacturers’ instructions (Exiqon, Vedbaek, Denmark). Briefly, cDNA was synthesized using a poly(T) primer and was amplified with locked nucleic acid (LNA) primers and SYBR Green master mix. Specific LNA PCR primer sets used were miRNA-31-5p and miRNA-149-5p. miRNA-218-5p was used as an endogenous control [17] and its expression was assessed in all samples. Those samples with a Ct value ≥ 30 for endogenous control were considered of low RNA quality and were excluded from the analysis (n=68). In the remaining samples, expression of miRNA-149-5p and miRNA-31-5p was analyzed. PCR reactions were carried out using specific miRNA protocol conditions provided by the manufacturers in an ABI7900HT instrument. At the end of the PCR cycles, melting curve analyses were performed.

### Data analysis

RT-qPCR data was analyzed using SDS 2.4 software (Applied Biosystems). An automatic baseline and a manual threshold of 2.0 were used for all miRNAs to record Cq values. Each experiment included a negative non-template control and an inter-experiment control. miRNA-218-5p was selected by using GeNorm as reference miRNA [46,47]. Relative expression levels of target miRNAs within a sample were expressed as ΔCt (Ct_miR218_ – Ct _target miRNA_).

### Statistical analysis

The probabilities of progression-free survival and cancer-specific survival were calculated using Kaplan-Meier curves. Statistical differences were identified by the log-rank test. Hazard ratios and their confidence interval were calculated. Spearman Test was used for correlations. In the multivariate analysis forward stepwise Cox regression was performed. Statistical significance was established at a p-value of 0.05, and accordingly 95% confidence intervals (CI) around hazard ratios (HR) are presented. SPSS 23.0 software was used for statistical analysis. Thereafter, Kaplan-Meier curves were generated. For this analysis, miRNA expression was dichotomized using the median value (-2.27) of miRNA-31-5p as a cut-off value.

### Pathway enrichment analysis

The DIANA-mirPath tool [48], using TargetScan as the target prediction algorithm, was used to identify the targets of miR-31-5p, and subsequent target enrichment analysis was performed in order to discover possible canonical altered pathways.

## SUPPLEMENTARY MATERIALS FIGURES AND TABLES



## Abbreviations

UTUC: upper urinary tract tumor
RNU: radical nephroureterectomy
