# Supplementary material for: Prognostic microRNAs in upper tract urothelial carcinoma: multicenter and international validation study
Source: Oncotarget. 2017 May 16;8(31):51522–9. doi: 10.18632/oncotarget.17884 (PMC5584265; doi:10.18632/oncotarget.17884)
Supplement: Supplementary file 1 [file oncotarget-08-51522-s001.pdf]

Prognostic microRNAs in upper tract urothelial carcinoma: multicenter and international validation study

SUPPLEMENTARY FIGURE AND TABLE

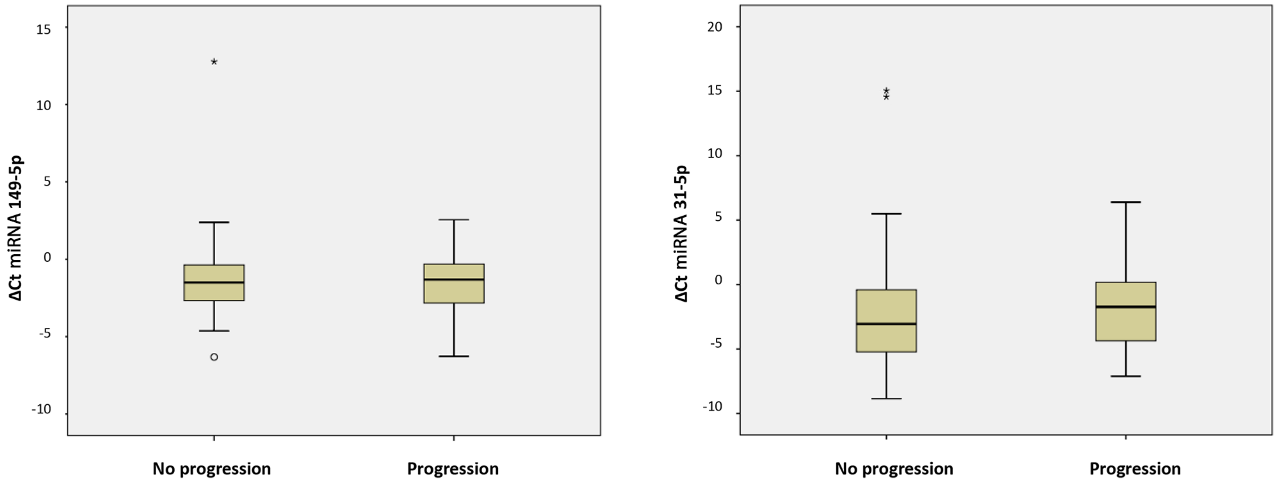

Supplementary Figure 1: Box plot of miRNA-149-5p and miRNA-31-5p expression pattern in progressing and non-progressing UTUC patients.

Supplementary Table 1: Altered predicted kyoto encyclopedia of genes and genomes pathways by miR-31-5p

KEGG pathway	miRNA-31-5p p-value	Genes involved
Thyroid hormone signaling pathway	0.0008	*MED12, ATP181, THRA, RCAN2, MED24, CCND1, HIF1A, ATP2A2, ATP1A1, RXRB and ACTB*
Steroid biosynthesis	0.0028	*DHCR24, CYP27B1*
Adherens junction	0.0089	*MET, VCL, RHOA, CTNNA1, PTPRJ, RAC1, MAP3K7, ACTB*
TNF signaling pathway	0.0100	*NFKB1, SELE, RPS6KA5, ICAM1, MAP3K14, MAPK9, MAPK8, CREB3L2, RIPK1, MAP3K7*
cGMP-PKG signaling pathway	0.0448	*CALM1, PPP1CC, GNA13, PDE3A, PPP3R1, ATP1B1, SLC25A5, RHOA, CALM2, PRKCE, IRS4, ITPR1, ITPR1, GNAQ, CREB3L2, ATP2A2, ATP1A1*
Inflammatory mediator regulation of TRP channels	0.0448	*IL1RAP, IL1R1, CALM1, PPP1CC, CALM2, PRKCE, MAPK9, MAPK8, PTGER2, ITPR1, GNAQ*
